# The crystal structure of 6-(4-chloro­phen­yl)-2-(4-methyl­benz­yl)imidazo[2,1-*b*][1,3,4]thia­diazole-5-carbaldehyde

**DOI:** 10.1107/S2056989016014754

**Published:** 2016-09-23

**Authors:** A. Sowmya, G. N. Anil Kumar, Sujeet Kumar, Subhas S. Karki

**Affiliations:** a Department of Physics, M. S. Ramaiah Institute of Technology, Bangalore, India; b Department of Pharmaceutical Chemistry, KLE University’s College of Pharmacy, Bangalore 560 010, India

**Keywords:** crystal structure, imidazo[2,1-*b*][1,3,4]thia­diazole, hydrogen bonding, C—H⋯π inter­actions

## Abstract

In the title imidazo[2,1-*b*][1,3,4]thia­diazole derivative, C_19_H_14_ClN_3_OS, the 4-methyl­benzyl and chloro­phenyl rings are inclined to the planar imidazo[2,1-*b*][1,3,4]thia­diazole moiety (r.m.s. deviation = 0.012 Å) by 64.5 (1) and 3.7 (1)°, respectively. The mol­ecular structure is primarily stabilized by a strong intra­molecular C—H⋯O hydrogen bond, leading to the formation of a pseudo-seven-membered *S*(7) ring motif, and a short intra­molecular C—H⋯N contact forming an *S*(5) ring motif. In the crystal, mol­ecules are linked by pairs of C—H⋯S hydrogen bonds, forming inversion dimers. The dimers are linked by C—H⋯O and C—H⋯π inter­actions, forming chains propagating along [110].

## Abstract 2

The title imidazo[2,1-*b*][1,3,4]thia­diazole derivative is non-planar, with the 4-methyl­benzyl and chloro­phenyl rings being inclined to the imidazo[2,1-*b*][1,3,4]thia­diazole ring system by 64.5 (1) and 3.7 (1)°, respectively.

## Chemical context   

The search for potential drugs to fight cancer and the design of mol­ecules with limited side effects, particularly to the immune system, is an emerging area of research. Imidazo[2,1-*b*][1,3,4]thia­diazole derivatives have been reported for their promising biological activities, and the most recent studies indicate their potential as anti­tumor agents (Karki *et al.*, 2011 ▸). However, active heterocyclic pharmacophores particularly at position 5 of the imidazo[2,1-*b*][1,3,4]thia­diazole moiety have shown significant activities; substitution of aldehydes at the 5-position resulted in an improvement of their anti­cancer activity (Kumar *et al.*, 2014 ▸), whereas a substituted phenyl group enhanced the anti-tubercular activity (Ramprasad *et al.*, 2015 ▸). In view of the above, we report herein on the synthesis and crystal structure of title imidazo[2,1-*b*][1,3,4]thia­diazole derivative.
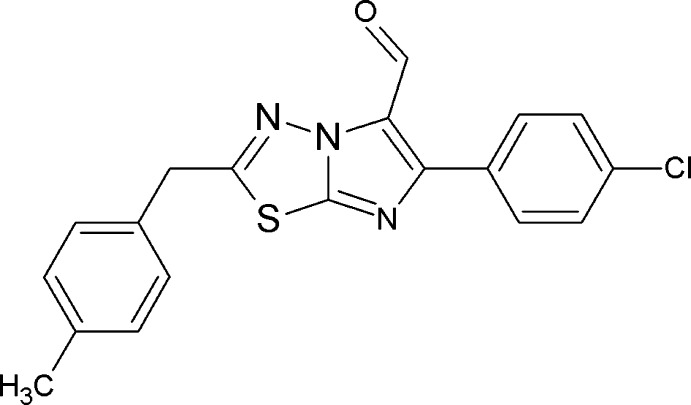


## Structural commentary   

**Figure 1:**
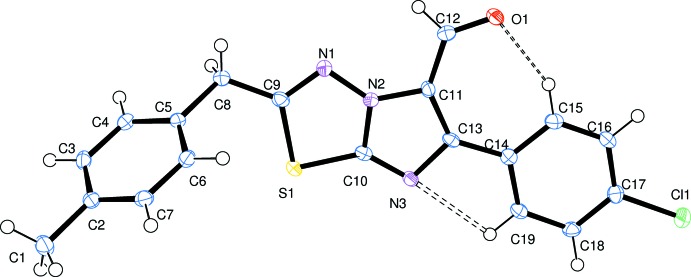
The mol­ecular structure of the title compound, showing the atom labelling. Displacement ellipsoids are drawn at 50% probability level. The intra­molecular inter­actions are shown as dashed lines (see Table 1 ▸).

The mol­ecular structure of the title compound is illustrated in Fig. 1 ▸. The carbaldehyde group is coplanar with the imidazo­thia­diazole ring system and *cis* to the chloro­phenyl ring. Bond C12=O1 is *cis* to the C13—C14 bond, which favours the formation of an intra­molecular C15—H15⋯O1 hydrogen bond (Table 1 ▸). The imidazole and thia­diazole rings show different π conjugations, resulting from their fused nature and also due to the groups attached to them. This is evident from the differences in the bond lengths S1—C9 [1.772 (4) Å] and S1—C10 [1.724 (2) Å] of the thia­diazole ring, indicating that the resonance effect caused by the imidazole ring is stronger than that caused by the thia­diazole ring. As a result, the imidazole system is more resonance stabilized. Additionally, the imidazo­thia­diazole moiety is planar and rigid with maximum deviations of 0.0182 (2) and −0.0078 (3) Å for atoms N2 and C13, respectively, from the mean plane. The 4-chloro­phenyl ring makes a dihedral angle of 3.7 (1)°, whereas the 4-methyl­benzyl ring is inclined at an angle of 64.5 (1)° with respect to the mean plane of the imidazo­thia­diazole ring system. The mol­ecular structure is primarily stabilized by the strong intra­molecular C15—H15⋯O1 hydrogen bond, leading to the formation of a pseudo-seven-membered hydrogen-bonded *S*(7) ring motif, and an intra­molecular C19—H19⋯N3 inter­action forming an *S*(5) ring motif, thus locking the mol­ecular conformation and eliminating conformational flexibility (Fig. 1 ▸ and Table 1 ▸).

## Supra­molecular features   

**Figure 2:**
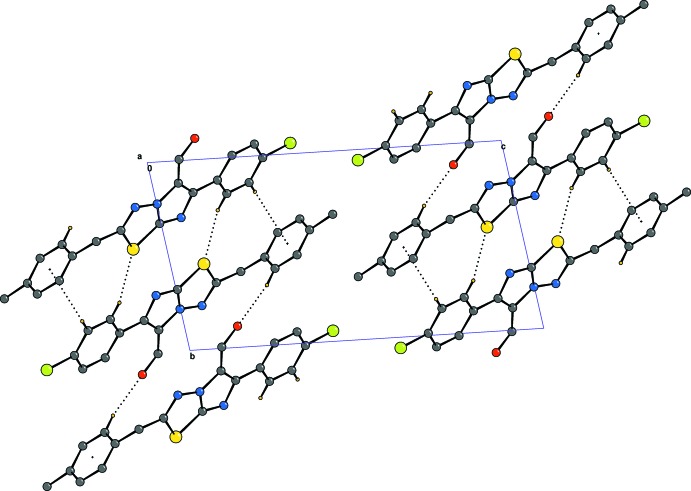
A view along the *a* axis of the crystal packing of the title compound. The inter­molecular inter­actions are shown as dashed lines (see Table 1 ▸) and, for clarity, H atoms not involved in these inter­actions have been omitted.

In the crystal, the solid-state structure is stabilized primarily by a pair of C—H⋯S hydrogen bonds, forming inversion dimers (Table 1 ▸ and Fig. 2 ▸). These dimers are linked by pairs of C—H⋯O hydrogen bonds and C—H⋯π inter­actions, forming chains propagating along [110]. There are no halogen inter­actions involving the chlorine atom, and no aromatic π–π stacking inter­actions present.

## Database survey   

A search of the Cambridge Structural Database (CSD, Version 5.37, last update May 2016; Groom *et al.*, 2016 ▸) gave 55 hits for mol­ecules containing the imidazo[2,1-*b*][1,3,4]thia­diazole moiety. A search for 2-benzyl-6-phenyl­imidazo[2,1-*b*][1,3,4]thia­diazo­les gave ten hits, and five of these compounds contain a 6-phenyl­imidazo[2,1-*b*][1,3,4]thia­diazole-5-carbaldehyde moiety. It is inter­esting to note that the aldehyde group generally accepts a hydrogen bond, and that the *para*-substituted halogens do not generate any significant weak inter­actions in the crystal packing, except for a C—H⋯F inter­action in 2-(4-fluoro­benz­yl)-6-phenyl­imidazo[2,1-*b*][1,3,4]thia­diazole-5-carbaldehyde (OWIFAC; Banu *et al.*, 2010 ▸), the 4-fluoro­benzyl analogue of the title compound.

## Synthesis and crystallization   

The title compound was obtained according to a reported procedure (Kumar *et al.*, 2014 ▸). The Vilsmeier reagent was prepared at 273–278 K by adding dropwise phospho­rous oxychloride (2.3 g, 15 mmol) into a stirred solution of DMF (10 ml). The 6-(4-chloro­phen­yl)-2-(4-methyl­benz­yl) imidazo[2,1-*b*][1,3,4]thia­diazole (4 mmol) was added slowly to the Vilsmeier reagent with stirring and cooling for 2 h. Further stirring was continued for 6 h at 353–363 K. The reaction mixture was then poured into 100 ml of water. The precipitate obtained was filtered, and neutralized with a cold aqueous solution of sodium carbonate. The solid obtained was filtered, washed with water and dried. Single crystals were obtained by slow evaporation of a solution in ethanol/DMF (2:1 *v*:*v*).

## Refinement   

Crystal data, data collection and structure refinement details are summarized in Table 2 ▸. H atoms were positioned geometrically, with N—H = 0.86 Å and C—H = 0.93–0.96 Å, and constrained to ride on their parent atoms with *U*_iso_(H) = 1.5*U*_eq_(C-meth­yl) and 1.2*U*_eq_(C,N) for other H atoms.

## Supplementary Material

Crystal structure: contains datablock(s) global, I. DOI: 10.1107/S2056989016014754/su5325sup1.cif

Structure factors: contains datablock(s) I. DOI: 10.1107/S2056989016014754/su5325Isup2.hkl

Supporting information file. DOI: 10.1107/S2056989016014754/su5325Isup3.cml

CCDC reference: 1504989

Additional supporting information: crystallographic information; 3D view; checkCIF report

## Figures and Tables

**Table 1:** Hydrogen-bond geometry (Å, °) *Cg* is the centroid of the C2–C7 ring.

*D*—H⋯*A*	*D*—H	H⋯*A*	*D*⋯*A*	*D*—H⋯*A*
C15—H15⋯O1	0.93	2.20	3.047 (3)	151
C19—H19⋯N3	0.93	2.42	2.788 (3)	103
C19—H19⋯S1^i^	0.93	2.83	3.733 (2)	165
C6—H6⋯O1^ii^	0.93	2.46	3.384 (3)	170
C18—H18⋯*Cg* ^i^	0.93	2.92	3.648 (12)	136

Symmetry codes: (i) 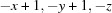; (ii) 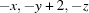.

**Table 2:** Experimental details

Crystal data	
Chemical formula	C_19_H_14_ClN_3_OS
*M*_r_	367.84
Crystal system, space group	Triclinic, *P* 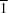
Temperature (K)	296
*a*, *b*, *c* (Å)	5.6138 (18), 9.018 (2), 16.514 (5)
α, β, γ (°)	80.533 (13), 87.519 (14), 83.353 (14)
*V* (Å^3^)	818.9 (4)
*Z*	2
Radiation type	Mo *K*α
μ (mm^−1^)	0.37
Crystal size (mm)	0.20 × 0.15 × 0.10

Data collection	
Diffractometer	Bruker *SMART* CCD area-detector
Absorption correction	Multi-scan (*SADABS*; Bruker, 2012 ▸)
*T*_min_, *T*_max_	0.941, 0.971
No. of measured, independent and observed [*I* > 2σ(*I*)] reflections	12059, 2966, 2530
*R*_int_	0.059
(sin θ/λ)_max_ (Å^−1^)	0.606

Refinement	
*R*[*F* ^2^ > 2σ(*F* ^2^)], *wR*(*F* ^2^), *S*	0.042, 0.110, 1.05
No. of reflections	2966
No. of parameters	228
H-atom treatment	H-atom parameters constrained
Δρ_max_, Δρ_min_ (e Å^−3^)	0.40, −0.26

Computer programs: *SMART* and *SAINT* (Bruker, 2012 ▸), *SHELXS97* (Sheldrick, 2008 ▸), *SHELXL2014* (Sheldrick, 2015 ▸), *ORTEP-3 for Windows* and *WinGX* (Farrugia, 2012 ▸), *CAMERON* (Watkin *et al.*, 1996 ▸), *Mercury* (Macrae *et al.*, 2008 ▸) and *PLATON* (Spek, 2009 ▸).

## supplementary crystallographic information

### Crystal data

**Table:**

C_19_H_14_ClN_3_OS	*Z* = 2
*M**_r_* = 367.84	*F*(000) = 380
Triclinic, *P*1	*D*_x_ = 1.492 Mg m^−^^3^
Hall symbol: -P 1	Mo *K*α radiation, λ = 0.71073 Å
*a* = 5.6138 (18) Å	Cell parameters from 1890 reflections
*b* = 9.018 (2) Å	θ = 3.3–26.4°
*c* = 16.514 (5) Å	µ = 0.37 mm^−^^1^
α = 80.533 (13)°	*T* = 296 K
β = 87.519 (14)°	Block, colourless
γ = 83.353 (14)°	0.20 × 0.15 × 0.10 mm
*V* = 818.9 (4) Å^3^	

### Data collection

**Table:**

Bruker SMART CCD area-detector diffractometer	2966 independent reflections
Radiation source: fine-focus sealed tube	2530 reflections with *I* > 2σ(*I*)
Graphite monochromator	*R*_int_ = 0.059
ω and φ scans	θ_max_ = 25.5°, θ_min_ = 1.3°
Absorption correction: multi-scan (SADABS; Bruker, 2012)	*h* = −6→6
*T*_min_ = 0.941, *T*_max_ = 0.971	*k* = −11→11
12059 measured reflections	*l* = −20→20

### Refinement

**Table:**

Refinement on *F*^2^	Hydrogen site location: inferred from neighbouring sites
Least-squares matrix: full	H-atom parameters constrained
*R*[*F*^2^ > 2σ(*F*^2^)] = 0.042	*w* = 1/[σ^2^(*F*_o_^2^) + (0.0537*P*)^2^ + 0.289*P*] where *P* = (*F*_o_^2^ + 2*F*_c_^2^)/3
*wR*(*F*^2^) = 0.110	(Δ/σ)_max_ = 0.001
*S* = 1.05	Δρ_max_ = 0.40 e Å^−^^3^
2966 reflections	Δρ_min_ = −0.26 e Å^−^^3^
228 parameters	Extinction correction: SHELXL2014 (Sheldrick, 2015)
0 restraints	Extinction coefficient: 0.015 (3)

### Special details

**Table:**

Geometry. All esds (except the esd in the dihedral angle between two l.s. planes) are estimated using the full covariance matrix. The cell esds are taken into account individually in the estimation of esds in distances, angles and torsion angles; correlations between esds in cell parameters are only used when they are defined by crystal symmetry. An approximate (isotropic) treatment of cell esds is used for estimating esds involving l.s. planes.

### Fractional atomic coordinates and isotropic or equivalent isotropic displacement parameters (Å^2^)

**Table:**

	*x*	*y*	*z*	*U*_iso_*/*U*_eq_	
S1	0.13628 (9)	0.55026 (6)	0.09398 (3)	0.01783 (18)	
Cl1	0.76563 (10)	1.05603 (6)	−0.41113 (3)	0.02857 (19)	
O1	−0.2181 (3)	1.11191 (15)	−0.14801 (9)	0.0188 (4)	
N1	−0.2122 (3)	0.76902 (19)	0.06190 (10)	0.0169 (4)	
N2	−0.0436 (3)	0.77957 (18)	−0.00135 (10)	0.0149 (4)	
N3	0.3040 (3)	0.69994 (18)	−0.05952 (10)	0.0158 (4)	
C1	0.2608 (4)	0.3316 (3)	0.48434 (13)	0.0254 (5)	
H1A	0.3880	0.3909	0.4910	0.038*	
H1B	0.3278	0.2319	0.4766	0.038*	
H1C	0.1580	0.3249	0.5325	0.038*	
C2	0.1165 (4)	0.4061 (2)	0.41000 (12)	0.0182 (5)	
C3	−0.0867 (4)	0.3460 (2)	0.38955 (13)	0.0194 (5)	
H3	−0.1379	0.2620	0.4234	0.023*	
C4	−0.2144 (4)	0.4091 (2)	0.31943 (12)	0.0170 (5)	
H4	−0.3490	0.3668	0.3070	0.020*	
C5	−0.1425 (4)	0.5349 (2)	0.26779 (12)	0.0164 (5)	
C6	0.0587 (4)	0.5973 (2)	0.28907 (13)	0.0187 (5)	
H6	0.1082	0.6825	0.2559	0.022*	
C7	0.1852 (4)	0.5335 (2)	0.35916 (13)	0.0183 (5)	
H7	0.3182	0.5768	0.3722	0.022*	
C8	−0.2861 (4)	0.6024 (2)	0.19192 (13)	0.0202 (5)	
H8A	−0.3853	0.5279	0.1800	0.024*	
H8B	−0.3928	0.6887	0.2045	0.024*	
C9	−0.1409 (4)	0.6527 (2)	0.11621 (12)	0.0171 (5)	
C10	0.1526 (4)	0.6762 (2)	0.00375 (12)	0.0159 (5)	
C11	−0.0221 (4)	0.8823 (2)	−0.07365 (12)	0.0152 (4)	
C12	−0.2119 (4)	1.0071 (2)	−0.09103 (13)	0.0174 (5)	
H12	−0.3429	1.0065	−0.0545	0.021*	
C13	0.1975 (4)	0.8283 (2)	−0.10865 (12)	0.0153 (5)	
C14	0.3268 (4)	0.8852 (2)	−0.18484 (12)	0.0157 (4)	
C15	0.2369 (4)	1.0111 (3)	−0.24086 (13)	0.0242 (5)	
H15	0.0863	1.0608	−0.2313	0.029*	
C16	0.3697 (4)	1.0622 (3)	−0.31028 (14)	0.0257 (5)	
H16	0.3084	1.1459	−0.3471	0.031*	
C17	0.5944 (4)	0.9882 (2)	−0.32487 (13)	0.0198 (5)	
C18	0.6866 (4)	0.8621 (2)	−0.27129 (13)	0.0185 (5)	
H18	0.8365	0.8122	−0.2816	0.022*	
C19	0.5524 (4)	0.8117 (2)	−0.20223 (13)	0.0171 (5)	
H19	0.6135	0.7266	−0.1663	0.021*	

### Atomic displacement parameters (Å^2^)

**Table:**

	*U*^11^	*U*^22^	*U*^33^	*U*^12^	*U*^13^	*U*^23^
S1	0.0166 (3)	0.0178 (3)	0.0168 (3)	0.0008 (2)	−0.0021 (2)	0.0024 (2)
Cl1	0.0260 (4)	0.0312 (3)	0.0236 (3)	0.0007 (2)	0.0075 (2)	0.0052 (2)
O1	0.0177 (8)	0.0161 (8)	0.0209 (8)	0.0003 (6)	−0.0028 (6)	0.0018 (6)
N1	0.0156 (10)	0.0195 (9)	0.0147 (9)	−0.0018 (7)	−0.0001 (7)	−0.0003 (7)
N2	0.0126 (10)	0.0169 (9)	0.0148 (9)	−0.0014 (7)	−0.0014 (7)	−0.0011 (7)
N3	0.0155 (10)	0.0146 (9)	0.0160 (9)	−0.0001 (7)	−0.0027 (7)	0.0002 (7)
C1	0.0276 (14)	0.0288 (12)	0.0171 (11)	0.0048 (10)	−0.0011 (10)	−0.0007 (9)
C2	0.0189 (12)	0.0200 (11)	0.0138 (10)	0.0065 (8)	0.0032 (9)	−0.0037 (8)
C3	0.0230 (13)	0.0156 (11)	0.0169 (11)	0.0009 (8)	0.0053 (9)	0.0013 (8)
C4	0.0155 (12)	0.0185 (11)	0.0171 (11)	−0.0013 (8)	0.0027 (9)	−0.0044 (8)
C5	0.0141 (12)	0.0188 (11)	0.0146 (10)	0.0026 (8)	0.0008 (8)	−0.0014 (8)
C6	0.0172 (12)	0.0172 (11)	0.0198 (11)	−0.0003 (8)	0.0041 (9)	0.0003 (9)
C7	0.0143 (12)	0.0217 (11)	0.0191 (11)	0.0002 (8)	0.0000 (9)	−0.0051 (9)
C8	0.0140 (12)	0.0238 (12)	0.0205 (11)	−0.0014 (8)	−0.0010 (9)	0.0026 (9)
C9	0.0148 (12)	0.0197 (11)	0.0169 (11)	−0.0012 (8)	−0.0045 (9)	−0.0024 (8)
C10	0.0151 (12)	0.0140 (10)	0.0183 (11)	−0.0002 (8)	−0.0033 (9)	−0.0022 (8)
C11	0.0162 (12)	0.0165 (10)	0.0123 (10)	−0.0026 (8)	−0.0017 (8)	0.0006 (8)
C12	0.0132 (12)	0.0197 (11)	0.0197 (11)	−0.0016 (8)	−0.0012 (9)	−0.0044 (9)
C13	0.0143 (11)	0.0153 (10)	0.0165 (11)	−0.0008 (8)	−0.0062 (8)	−0.0024 (8)
C14	0.0166 (12)	0.0156 (10)	0.0157 (10)	−0.0027 (8)	−0.0027 (8)	−0.0038 (8)
C15	0.0180 (13)	0.0289 (13)	0.0216 (12)	0.0060 (9)	0.0020 (9)	0.0015 (10)
C16	0.0210 (13)	0.0272 (12)	0.0227 (12)	0.0059 (9)	−0.0001 (10)	0.0079 (9)
C17	0.0212 (13)	0.0228 (11)	0.0149 (11)	−0.0023 (9)	0.0003 (9)	−0.0015 (9)
C18	0.0157 (12)	0.0181 (11)	0.0212 (11)	0.0027 (8)	0.0010 (9)	−0.0049 (9)
C19	0.0181 (12)	0.0133 (10)	0.0193 (11)	0.0007 (8)	−0.0033 (9)	−0.0017 (8)

### Geometric parameters (Å, º)

**Table:**

S1—C10	1.724 (2)	C5—C8	1.521 (3)
S1—C9	1.772 (2)	C6—C7	1.390 (3)
Cl1—C17	1.749 (2)	C6—H6	0.9300
O1—C12	1.218 (2)	C7—H7	0.9300
N1—C9	1.299 (3)	C8—C9	1.498 (3)
N1—N2	1.378 (2)	C8—H8A	0.9700
N2—C10	1.355 (3)	C8—H8B	0.9700
N2—C11	1.395 (3)	C11—C13	1.408 (3)
N3—C10	1.323 (3)	C11—C12	1.458 (3)
N3—C13	1.389 (2)	C12—H12	0.9300
C1—C2	1.518 (3)	C13—C14	1.472 (3)
C1—H1A	0.9600	C14—C19	1.401 (3)
C1—H1B	0.9600	C14—C15	1.400 (3)
C1—H1C	0.9600	C15—C16	1.383 (3)
C2—C7	1.390 (3)	C15—H15	0.9300
C2—C3	1.395 (3)	C16—C17	1.388 (3)
C3—C4	1.393 (3)	C16—H16	0.9300
C3—H3	0.9300	C17—C18	1.384 (3)
C4—C5	1.391 (3)	C18—C19	1.382 (3)
C4—H4	0.9300	C18—H18	0.9300
C5—C6	1.401 (3)	C19—H19	0.9300
			
C10—S1—C9	87.97 (10)	H8A—C8—H8B	107.5
C9—N1—N2	108.08 (16)	N1—C9—C8	122.86 (19)
C10—N2—N1	118.52 (17)	N1—C9—S1	116.07 (16)
C10—N2—C11	108.08 (17)	C8—C9—S1	121.03 (15)
N1—N2—C11	133.36 (17)	N3—C10—N2	113.00 (18)
C10—N3—C13	104.36 (16)	N3—C10—S1	137.61 (15)
C2—C1—H1A	109.5	N2—C10—S1	109.37 (15)
C2—C1—H1B	109.5	N2—C11—C13	103.37 (17)
H1A—C1—H1B	109.5	N2—C11—C12	117.73 (19)
C2—C1—H1C	109.5	C13—C11—C12	138.89 (19)
H1A—C1—H1C	109.5	O1—C12—C11	127.2 (2)
H1B—C1—H1C	109.5	O1—C12—H12	116.4
C7—C2—C3	117.96 (19)	C11—C12—H12	116.4
C7—C2—C1	121.3 (2)	N3—C13—C11	111.17 (18)
C3—C2—C1	120.7 (2)	N3—C13—C14	117.41 (18)
C2—C3—C4	121.3 (2)	C11—C13—C14	131.42 (18)
C2—C3—H3	119.4	C19—C14—C15	117.89 (19)
C4—C3—H3	119.4	C19—C14—C13	118.83 (18)
C5—C4—C3	120.6 (2)	C15—C14—C13	123.27 (19)
C5—C4—H4	119.7	C16—C15—C14	120.7 (2)
C3—C4—H4	119.7	C16—C15—H15	119.6
C4—C5—C6	118.18 (19)	C14—C15—H15	119.6
C4—C5—C8	119.60 (19)	C15—C16—C17	119.9 (2)
C6—C5—C8	122.20 (19)	C15—C16—H16	120.1
C7—C6—C5	120.8 (2)	C17—C16—H16	120.1
C7—C6—H6	119.6	C18—C17—C16	120.8 (2)
C5—C6—H6	119.6	C18—C17—Cl1	119.38 (17)
C2—C7—C6	121.1 (2)	C16—C17—Cl1	119.84 (16)
C2—C7—H7	119.4	C19—C18—C17	118.9 (2)
C6—C7—H7	119.4	C19—C18—H18	120.5
C9—C8—C5	115.50 (18)	C17—C18—H18	120.5
C9—C8—H8A	108.4	C18—C19—C14	121.79 (19)
C5—C8—H8A	108.4	C18—C19—H19	119.1
C9—C8—H8B	108.4	C14—C19—H19	119.1
C5—C8—H8B	108.4		
			
C9—N1—N2—C10	−0.5 (2)	C9—S1—C10—N2	−0.02 (15)
C9—N1—N2—C11	−177.9 (2)	C10—N2—C11—C13	0.5 (2)
C7—C2—C3—C4	1.4 (3)	N1—N2—C11—C13	178.12 (18)
C1—C2—C3—C4	−177.03 (18)	C10—N2—C11—C12	−178.46 (17)
C2—C3—C4—C5	−0.2 (3)	N1—N2—C11—C12	−0.9 (3)
C3—C4—C5—C6	−1.1 (3)	N2—C11—C12—O1	175.43 (19)
C3—C4—C5—C8	−179.56 (19)	C13—C11—C12—O1	−3.1 (4)
C4—C5—C6—C7	1.1 (3)	C10—N3—C13—C11	0.1 (2)
C8—C5—C6—C7	179.56 (19)	C10—N3—C13—C14	179.35 (17)
C3—C2—C7—C6	−1.4 (3)	N2—C11—C13—N3	−0.4 (2)
C1—C2—C7—C6	177.07 (19)	C12—C11—C13—N3	178.2 (2)
C5—C6—C7—C2	0.1 (3)	N2—C11—C13—C14	−179.49 (19)
C4—C5—C8—C9	−139.6 (2)	C12—C11—C13—C14	−0.8 (4)
C6—C5—C8—C9	41.9 (3)	N3—C13—C14—C19	−2.5 (3)
N2—N1—C9—C8	−177.12 (17)	C11—C13—C14—C19	176.5 (2)
N2—N1—C9—S1	0.5 (2)	N3—C13—C14—C15	178.33 (18)
C5—C8—C9—N1	−146.1 (2)	C11—C13—C14—C15	−2.7 (3)
C5—C8—C9—S1	36.4 (3)	C19—C14—C15—C16	−1.1 (3)
C10—S1—C9—N1	−0.27 (17)	C13—C14—C15—C16	178.0 (2)
C10—S1—C9—C8	177.37 (18)	C14—C15—C16—C17	0.0 (4)
C13—N3—C10—N2	0.2 (2)	C15—C16—C17—C18	0.9 (3)
C13—N3—C10—S1	−178.12 (18)	C15—C16—C17—Cl1	−178.23 (18)
N1—N2—C10—N3	−178.50 (16)	C16—C17—C18—C19	−0.8 (3)
C11—N2—C10—N3	−0.5 (2)	Cl1—C17—C18—C19	178.40 (15)
N1—N2—C10—S1	0.3 (2)	C17—C18—C19—C14	−0.4 (3)
C11—N2—C10—S1	178.32 (13)	C15—C14—C19—C18	1.3 (3)
C9—S1—C10—N3	178.4 (2)	C13—C14—C19—C18	−177.90 (18)

### Hydrogen-bond geometry (Å, º)

Cg is the centroid of the C2–C7 ring.

**Table:**

*D*—H···*A*	*D*—H	H···*A*	*D*···*A*	*D*—H···*A*
C15—H15···O1	0.93	2.20	3.047 (3)	151
C19—H19···N3	0.93	2.42	2.788 (3)	103
C19—H19···S1^i^	0.93	2.83	3.733 (2)	165
C6—H6···O1^ii^	0.93	2.46	3.384 (3)	170
C18—H18···*Cg*^i^	0.93	2.92	3.648 (12)	136

Symmetry codes: (i) −*x*+1, −*y*+1, −*z*; (ii) −*x*, −*y*+2, −*z*.

## References

[bb1] Banu, A., Lamani, R. S., Khazi, I. M. & Begum, N. S. (2010). *Mol. Cryst. Liq. Cryst.* **533**, 141–151.

[bb2] Bruker (2012). *APEX2*, *SAINT* and *SADABS*. Bruker AXS Inc., Madison, Wisconsin, USA.

[bb3] Farrugia, L. J. (2012). *J. Appl. Cryst.* **45**, 849–854.

[bb4] Groom, C. R., Bruno, I. J., Lightfoot, M. P. & Ward, S. C. (2016). *Acta Cryst.* B**72**, 171–179. 10.1107/S2052520616003954 PMC4822653 27048719

[bb5] Karki, S. S., Panjamurthy, K., Kumar, S., Nambiar, M., Ramareddy, S. A., Chiruvella, K. K. & Raghavan, S. C. (2011). *Eur. J. Med. Chem.* **46**, 2109–2116. 10.1016/j.ejmech.2011.02.064 21439690

[bb6] Kumar, S., Hegde, M., Gopalakrishnan, V., Renuka, V. K., Ramareddy, S. A., De Clercq, E., Schols, D., Gudibabande Narasimhamurthy, A. K., Raghavan, S. C. & Karki, S. S. (2014). *Eur. J. Med. Chem.* **84**, 687–697. 10.1016/j.ejmech.2014.07.054 25064346

[bb7] Macrae, C. F., Bruno, I. J., Chisholm, J. A., Edgington, P. R., McCabe, P., Pidcock, E., Rodriguez-Monge, L., Taylor, R., van de Streek, J. & Wood, P. A. (2008). *J. Appl. Cryst.* **41**, 466–470.

[bb8] Ramprasad, J., Nayak, N., Dalimba, U., Yogeeswari, P., Sriram, D., Peethambar, S. K., Achur, R. & Kumar, H. S. S. (2015). *Eur. J. Med. Chem.* **95**, 49–63. 10.1016/j.ejmech.2015.03.024 25794789

[bb9] Sheldrick, G. M. (2008). *Acta Cryst.* A**64**, 112–122. 10.1107/S0108767307043930 18156677

[bb10] Sheldrick, G. M. (2015). *Acta Cryst.* C**71**, 3–8.

[bb11] Spek, A. L. (2009). *Acta Cryst.* D**65**, 148–155. 10.1107/S090744490804362X PMC2631630 19171970

[bb12] Watkin, D. J., Prout, C. K. & Pearce, L. J. (1996). *CAMERON*. Chemical Crystallography Laboratory, Oxford, England.

